# Cardiovascular medicine at face value: a qualitative pilot study on clinical axiology

**DOI:** 10.1186/1747-5341-8-3

**Published:** 2013-03-27

**Authors:** Adalberto de Hoyos, Rodrigo Nava-Diosdado, Jorge Mendez, Sergio Ricco, Ana Serrano, Carmen Flores Cisneros, Carlos Macías-Ojeda, Héctor Cisneros, David Bialostozky, Nelly Altamirano-Bustamante, Myriam M Altamirano-Bustamante

**Affiliations:** 1 Grupo transfuncional en ética clínica, Centro Médico Nacional Siglo XXI, IMSS, Av. Cuauhtémoc 330 Col. Doctores, México, 06720, D.F Mexico; 2 FES Acatlán, Universidad Nacional Autónoma de México, Av. Cuauhtémoc 330 Col. Doctores, México, 06720, D.F. Mexico; 3 Universidad Pedagógica Nacional, Mexico, Mexico; 4 Instituto Nacional de Perinatología, Mexico, Mexico; 5 Universidad Anáhuac, Mexico, Mexico; 6 Facultad de Ciencias, UNAM, Mexico, Mexico; 7 Instituto Nacional de Pediatría, Secretaría de Salud, Mexico, Mexico; 8 Unidad de Investigación de Enfermedades Metabólicas, Centro Médico Nacional Siglo XXI, IMSS, Av. Insurgentes 3300. Copilco Universidad, México, 04530, D.F. Mexico; 9 Instituto Nacional de Cardiología, Ignacio Chávez, Mexico, Mexico

**Keywords:** Bioethics, Qualitative analysis, Patient-doctor relationship, Values, Cardiology, Ethical dilemmas

## Abstract

**Introduction:**

Cardiology is characterized by its state-of-the-art biomedical technology and the predominance of Evidence-Based Medicine. This predominance makes it difficult for healthcare professionals to deal with the ethical dilemmas that emerge in this subspecialty. This paper is a first endeavor to empirically investigate the axiological foundations of the healthcare professionals in a cardiology hospital. Our pilot study selected, as the target population, cardiology personnel not only because of their difficult ethical deliberations but also because of the stringent conditions in which they have to make them. Therefore, there is an urgent need to reconsider clinical ethics and Value-Based Medicine. This study proposes a qualitative analysis of the values and the virtues of healthcare professionals in a cardiology hospital in order to establish how the former impact upon the medical and ethical decisions made by the latter.

**Results:**

We point out the need for strengthening the roles of healthcare personnel as educators and guidance counselors in order to meet the ends of medicine, as well as the need for an ethical discernment that is compatible with our results, namely, that the ethical values developed by healthcare professionals stem from their life history as well as their professional education.

**Conclusion:**

We establish the kind of actions, communication skills and empathy that are required to build a stronger patient-healthcare professional relationship, which at the same time improves prognosis, treatment efficiency and therapeutic adhesion.

## Introduction

Cardiology is one of the subspecialties in which evidence-based medicine (EBM) is predominant. Due to the combination of medical expertise and the use of imaging (SPECT gate, Angio TAC, PET, NMR and Echocardiography), cardiology is capable of great diagnostic security and objectivity. Cardiology offers efficient diagnostic, therapeutic and prognostic results ensuring life quality for the patient [1]. However, it presents ethical dilemmas that are very unlikely to be solved solely based on the available clinical evidence. The ethical dilemmas specific to this subspecialty are well known; they include, among others, issues arising from the decisions to be made between what can be done and what should done related to chronic ischemic cardiopathy, acute coronary ischemic syndrome. Ethical deliberation is needed to decide whether to resurrect a patient in the case of a heart attack or malignant arrhythmia, [2,3], the use of scarce resources in the case of organ transplantation [4], the process of informed consent and the role of the living will [5]. In view of these dilemmas, an urgent necessity arises in cardiology to reinforce the pairing of values-based medicine (VBM) and EBM.

Consider an informed consent dilemma such as the one in Altamirano *et al*. [6], where a girl suffered from several congenital cardiopathies having undergone, during her childhood, several interventions, such as a patent ductus arteriosus (PDA) ligation, and an atrial septal defect (ASD) repair, among others. At age 16 she became pregnant, having class II NYHA^a^ cardiac insufficiency. A therapeutic abortion was recommended to preserve and not overload cardiac function, but the patient refused the proposed treatment. It is very complicated to approach a case such as this one, exclusively from the EBM point of view.

If treatment is rejected, the patient should not be left alone to face the consequences of her choices. Instead, new alternatives should be sought, taking into account the personal, social and cultural context of the patient. It is also important to take into account the communication skills of the healthcare personnel dealing with the patient as a human being, and not only as a sick body. A strategy allowing for the EBM and VBM paradigms to work together offers a better opportunity to solve such dilemmas.

EBM was developed in the 90s with a positivist vision of the biomedicine and focuses on treatment of disease, whereas VBM is patient-centered and has more of a bio-psycho- social approach, combining the ethical values of medical professionals with the patient’s interests, values and form of life [1,2,7]. We posit that communication between EBM and VBM is one of the greater challenges facing contemporary medical practice [3].

Values are normative systems that allow us to consider the priority, the convenience or desirability of a course of action with aims to certain ends [8]. Therefore, if providing medical assistance is considered to be more than a technical or epistemological task then values, other than scientific ones, have to be considered when treating a patient. Ethical, economic, social, and even political values influence the actions of healthcare professionals when facing medical practice dilemmas.

A first step in integrating clinical ethics and values-based medicine (VBM) into cardiology is to study the final purposes of healthcare along with the values linked to them [9,10]. These values must allow healthcare professionals to carefully reflect on their practice in order to properly address the problems that arise [11]. Physicians and healthcare professionals are continually making ethical decisions and have lost the habit of critical reflection. In cardiology, both diagnosis and treatment are carried out from one study to the next, guided by the interpretation of the illness that is being examined. This procedure is carried out without any participation from the patient; informed consent is often required only as a routine.

Many times, physicians seldom stop to think about their patients’ worries or fears. VBM considers the patient as a co-participant and as co-responsible in the decision making process. From this perspective, to carry out diagnosis and to provide the necessary treatment, it is important to consider the patient’s capabilities and social networks, together with the clinical data. VBM emphasizes clinical ethics, where the encounter between patient and healthcare personnel involves both technical and ethical considerations [12]. Pellegrino states that to deliberate about the rightness of a medical intervention, healthcare personnel must take into consideration the fulfillment of some general ends of medicine: healing, curing and caring for the patient [13]. Having specific ends of a professional role creates particular ethical obligations, and also requires special reflection about them [10].

In this paper we examine life history values and their relationship to the roles and virtues of a group of health professionals. We also examine the considerations addressed when faced with ethical dilemmas. Medical personnel often balance values and virtues in order to reach the goals of their practice; namely, the well-being of their patients.

Several ethical theories nourish VBM: For example, principlism and virtue ethics systematize and promote ethical deliberation in medical practice [4,7,13-15]. But a focus on values attempts to reach a wider phenomenon; alongside the ends, principles and traits of character that a physician should have, consideration is also given to the social dimensions and state of affairs that are valuable for medical practice. Another feature of VBM is the responsibility that healthcare professionals have when dealing with people from different cultural backgrounds. Since patients’ values and forms of life are not homogenous, healthcare professionals need to develop cultural competence and be able to stimulate intercultural’ dialogue, thus improving the encounter with patients [16].

Now, more than ever, EBM requires the participation of the patient in her own treatment and traditional clinical ethical theories such as principlism can be somewhat one sided, taking into consideration only the healthcare personnel’s perspective [14]. Virtue ethics may be more balanced, and we often use its theoretical framework in VBM [9,17]. But not all values are virtues. A virtue is concerned with the traits of character found and developed in the moral agent, while a value refers to a wider set of attitudes that guide action, as well as the states of affairs favorable in reaching certain goals. Therefore, in the overall picture of the encounter between patient and healthcare professional, our analysis considers the values and not exclusively the virtues, which may allow a better balance in the clinical relationship [18].

At a certain level, questions about values need to be answered from the perspective of the healthcare personnel. Quantitative methods are not well suited to reach the first person point of view that is needed; instead, we propose a qualitative method to analyze the perceptions that healthcare professionals have about their own practices. Qualitative analysis focuses on rigorous sampling and systematization, enabling these representations and their contexts to be studied in greater depth, even if taken from only a few cases. A pilot study will therefore enable an initial interpretation based upon the data gathered at interviews.

Toward this goal, a semi-structured interview guide was prepared to inquire into the priorities of healthcare professionals and the way they care for their patients [19,20]. The axiological analysis of the data took into account Schwartz’s methodological proposals [21], modified for a Mexican Population by Arciniega and González, using the Work Values Scale (EVAT 30) [22,23], which allowed us to define the axiological priorities of the healthcare personnel. As well, we adopted the views of Oakley & Cocking [10] and Pellegrino [9], regarding the virtues and vices of healthcare specialists.

This is a cross-functional study, an amalgam of perspectives from philosophy, anthropology and medicine, which attempts to capture the vision of each health specialist’s values in their everyday work. This study focuses on the representations^b^ that are specific to the medical professions. Beliefs, desires, meanings and their interactions structure representations of the world and the events in our environment. These representations allow us to value and choose certain courses of action over others.

Given that in cardiology treatment often requires changes in the patient’s lifestyle, it is necessary for healthcare professionals in this specialty to fulfill roles other than that of merely the provider of medical treatment, and in this way serve as educators and guidance counselors. These professional roles are important to achieving the ends of medicine that procure healing, curing and caring [13]. This empirical qualitative study was conducted at a level three cardiology hospital in Mexico City, and it explores these roles and the values associated with them from social, life history and professional perspectives. The results of this pilot study form a starting point in understanding the axiological configuration of cardiologic medicine in Mexico.

We believe that knowledge of the axiology of this clinical practice will contribute to fostering both EBM and VBM, thereby improving patient care through a deeper ethical regard for patients’ values and interests [11,18], without neglecting rigorous medical practice in the scientific arena.

## Methods

This work empirically studies the axiological representation of the health care professionals; it is the first step of a broader project on “Strengthening of Values Based Medicine: An on- line Educative Intervention in Clinical Ethics”, from Mexico’s Social Security Institute (IMSS), which had as its general objective the pairing of VBM-EBM through the nationwide promotion of an ethical culture in medicine [18,24].

As stated, the analysis was carried out at a level three cardiology hospital. The 4 interviewees were randomly selected from the 60 participants in the course from a third level cardiology hospital. Data were collected until theoretical saturation was reached [25,26]. The inclusion criteria for the informants’ selection were: a) those specialists who had concluded the pilot study in clinical ethics with a passing grade, b) participants who had obtained a diploma for the course, and c) health professionals who were willing to participate in a semi-structured interview.

The research ethics committee of the IMSS approved this study, and all of the participants received written and oral information on the project. Afterwards, participants were provided a written a letter of informed consent which they signed, and which insured the anonymity of all personal data. The letter of informed consent granted the authors of this project permission to use and to publish the data and results of this study.

These semi-structured, face to face, interviews were conducted by anthropologists focusing on building empathy and trust. The interviews were digitally recorded, and literal transcripts were made, while the codifications and analytical processes were conducted, along with feedback processes, among the members of the clinical ethics cross-functional team, which included anthropologists, philosophers, bioethicists, and physicians. One hundred codes were prepared, referring to the different subjects to be dealt with in the semi-structured interviews. These codes were grouped as follows: life history, workday, ethical discernment, patient-doctor relationship, medical procedures, decision taking, ethics committee and future expectations of the healthcare personnel (Table 1). The narratives were analyzed according to the method of Little *et al*. [27]. Data were stored, organized and coded using Atlas.ti 6.2 Software.

**Table 1:** Topics and values surveyed in the interviews*

**Life history**	**Ethical discernment**	**Patient-healthcare professional relationship**
**Openness to change:**	**Values:**	**Medical roles**
Self-direction	Autonomy	Medical assistance
Motivation	Justice	Education
	Benevolence	Counseling
**Conservation:**	Confidentiality	
Tradition	Compassion	**Professional virtues**
Conformity	Trust	Integrity
Security	Respect	Justice
		Prudence
**Self-transcendence:**	**Impact of medical attention:**	Moderation
Universalism	Life care	Self-effacement
Benevolence	Compliance with regulations	Strength
	Therapeutic utility	Patience
**Self-enhancement:**	Imminent death risk	
Achievement	Appreciate situations and consequences	**Interpersonal values**
Authority		Compassion
	**Anti-values**	Trust
	Heteronomy	Self-control
	Injustice	Self-effacement
	Unaccountability	
	Indifference	**Professional competence**
	Disrespectfulness	Efficiency in diagnostic
	Malfeasance	Efficiency in treatment
	Discrimination	Problem resolution

* The bold subtitles refer to the different topics surveyed in the interviews and each topic encompasses different values.

## Results

### Life history

We traced the values that prevail throughout the life history of the interviewees and examined their relationship to healthcare professions. We found that the high scoring values (openness to change, conservation, self-transcendence and self-enhancement) are often balanced and adjusted to attain the goals of the clinical practice. For example, *openness to change*, characterized by values such as *self*-*direction* and *motivation*[21], featured independent decision-making, innovation and challenge. In some cases, this openness to change was expressed by a willingness to promote improvements in local medical models that would bolster positive changes in the workplace:However, the *conservation* category, which encompasses values such as *conformity, security, and tradition,* presents life and professional values’ bias toward preserving harmony and stability in different social relationships. The answers linked to *conformity* were more frequent than those linked to *openness to change* and they were the dominant values in the life history of the interviewees.

I think that a doctor should be able to select his patient and a patient his doctor; it would be great if conditions were like they are in Canada, where you can choose. It would be good if going to the doctor were a decision and not a punishment. (Medical specialist 1)

*Conformity* describes a passive attitude; even when a possibility for improvement is perceived, there is a persistence of actions dictated by tradition. For example, a medical specialist states:

Institutional time constraints don’t let us, it is one of the absurdities we have here, […] I think we could improve things, but I honestly don’t see it happening. (Medical specialist 1)

We have seen that family tradition plays an important role in the choice of these professions. Vocation is influenced by the social and cultural environment in which values and beliefs are shared, as illustrated by this comment by another medical specialist:

My attraction towards medicine comes from the social and medical activity in my town… the role played by the doctor and the priest in a small town is very important. (Medical specialist 2)

Other opposing value categories considered in the life history were those of self-transcendence and self-enhancement. In Schwartz’s [21] bi-dimensional structure, *self-transcendence* encompasses values such as *universalism* and *benevolence,* which involve an appreciation and protection of the people one comes in contact with, and a strong consideration for the well-being of others. For example, a medical specialist stated:

[G]uiding him in reintegrating into his personal, work, and family life, into his context, since in here he feels a bit like an invalid. We must help him in his physical rehabilitation and with his self-confidence, so he can reintegrate into daily life. (Medical specialist 3)

On the other hand, the *self-enhancement* category is opposite to that of *self-transcendence* category*,* and includes values such as *authority* and *achievement,* providing prestige and social status, control of resources, and personal success. In this category, we observed answers in which the clinicians’ personal well-being was placed above that of the people with whom she is frequently in contact. For example:

When studying medicine, I wanted to be at a level three hospital, I think I have achieved that, I think I have achieved part of those expectations. (Medical specialist 3)

Our results show that these attitudes and their corresponding values are well balanced and appear frequently in the perceptions of healthcare professionals, who express feelings of satisfaction at having carried out their duties successfully for the benefit of others, while also having achieved personal or professional goals.

### Healthcare roles

The three roles that healthcare personnel must play in order to fulfill the ends of medicine are: (i) as *a providers of medical assistance, (ii) as guidance* counselors and (iii) *as educators*[28]. These roles include helping patients deal with illness in everyday life through treatments, medical procedures, and prescriptions, as well as providing indications on how to react when faced with illness. Our results showed that in their representations, professionals in the field of cardiology are constantly attentive to their roles as providers of medical assistance and guidance counselors.

The following is an example of a description given by a specialist regarding the *medical assistance* provided:

I practice at both ends of cardiology, I don’t like the middle area, I practice preventive cardiology, patients without heart attack problems, diabetics, people with high blood pressure, generally young people, and at the other end are people with risk of heart failure. This is a professional deviation because my first years as a cardiologist were spent in a heart failure residency; a lot of people don’t like this because these patients take up too much time, for one thing, and for another, they don’t bring in a lot of money. (Medical Specialist 1)

A significant aspect of the specialists’ answers focused on *guidance*, as being crucial in promoting a close relationship with patients, in that it helps patients to anticipate future scenarios and make decisions accordingly:

One may warn them, not reprehend them. Who am I to reprehend a patient? His decision has to be respected, although one must emphasize on the consequences that this decision can have. (Medical specialist 1)

However, we found that the role of *educator* was mentioned to a lesser extent. In the educator role, specialists pass on their knowledge in a clear and precise way, enabling patients to follow directions and change their habits in an informed manner:

They are content and satisfied because we don’t use technical terms, but we do explain why we are taking a sample. (Laboratory personnel)

In our pilot group, the predominance of medical assistance and guidance roles was clear. In contrast, the educator role was absent. We argue that all three roles are needed to effectively and ethically meet the ends of medicine. These roles reflect and describe the encounter between patient and healthcare personnel. Much of the literature on clinical ethics has focused on the values and virtues of ethical discernment and decision making process in this relationship. Therefore, we assert that the actualization of these three roles is vital to establishing and sustaining the qualities necessary for approaching, apprehending, and articulating ethical judgments in clinical care.

### Ethical discernment

This study was primarily focused on representations in two areas: (i) the *impact of medical attention* and (ii) the *values* of the therapeutic relationship between the patient and healthcare professionals. In (i) *the impact of medical attention* category, we studied the main concerns of these professionals when seeing a patient. The most frequent answers related to *life care* and the *capacity to appreciate situations and their consequences*. Regarding their ethical deliberation, healthcare professionals alluded to these factors. For example, on providing treatment:

Right now I have patients with clear indications for surgery. I have not sent them in, and I don´t intend to send in some of them because there is a high risk of complications, of suffering more with the treatment than without it. Without the treatment, there's likelihood of sudden death, with the surgery, the patient might not die, but will live miserably at this age. (Medical specialist 3)

The second area in which ethical discernment was studied was the specific configuration of (ii) the *values* of the therapeutic relationship between the patient and healthcare professionals. In this category we found a clear concern for *justice*, as the value with the highest incidence in clinicians’ ethical discernments:

They must be treated equally and never be feared or anything, I have attended many courses on how to treat patients with AIDS. Many people are afraid of them and treat them badly; […] everybody will get one illness or another, so I don’t reject anybody. (Medical specialist 3)

*Respect* is another value that was considered fundamental in the patient-doctor relationship:

One must give priority to seeing patients and to answering the questions they may have, give them proper guidance, respect them, communicate with them as soon as possible, and if there is already direct contact with the patient, to consider him the highest priority regardless of how small his request, question or his ailment is. (Medical specialist 2)

Medical *professional virtues* were also studied within the patient-doctor relationship. The virtues surveyed were those that Pellegrino had proposed as essential to the fulfillment of the ends of medicine [13]. We found that the professional virtues more often revealed by the interviewees were: *patience*- the ability to know how to wait and be tolerant; *self-effacement*- the modesty and an acknowledgement of one’s limitations, *justice-* as virtue requiring an equal level of medical attention to specific patients, but also providing the general population with access to healthcare; and finally *prudence-* identifying and executing correct behavior in difficult situations, for the right reasons.

We hold that descriptions of these virtues in medicine reveal specific traits of the cardiology specialists’ professional practice. The results reveal a clinical practice where self-effacement is held in high regard:

… We can never be completely content with what we are doing, with what we have done… we have to be honest about our limitations. (Medical specialist 3)

Even so, *prudence* and *justice* also frequently and consistently appear in the interviewees’ responses:

We’ve had extremely restless or old patients that want to leave and try to get up from the bed, who knows how they do it, they are tied down, but they fight and fight, and that hurts them, that’s why it is convenient to sedate them, but all that is explained to the patients and their family members. (Laboratory personnel)

*Interpersonal values* in cardiology shape the practices that direct the healthcare provider toward presentation, emotions and dealings with the patient. Our results show that the most significant of these values were: *compassion, self-effacement,* and *trust. Compassion* is seen as caring for the patient, hoping for her improvement, or at least for her relief from suffering:

… Poor people, they’re already here, uncomfortable, tired… many of them seem almost forsaken, but no, they leave; when we see them again, they’ve moved to an outpatient service! Good, he’s moved to an outpatient service! He’ll recover… (Laboratory personnel)

## Discussion

The myriad problems arising in clinical practice have been attributed to a decline of professionalism brought about by factors such as dissatisfaction, burn-out, and attrition [29,30]. While these factors are influential it is also crucial to focus upon healthcare professionals’ values, relative to professional attitudes and conduct.

Values guide actions and objectives in both daily life and in the professional environment [31]. Exploring these values, as well as key professional virtues was important in our forging connections between Schwartz’s [21] theory of values, Oakley & Cocking’s proposal [10], and Pellegrino’s claims regarding the virtues of and in medicine [9]. Herein, we have developed an exploratory study on the values of healthcare personnel in a cardiology hospital as a community. We have not taken into consideration a gender perspective, even though both men and women are important parts of this community. Such a perspective surpasses the objectives and scope of this paper.

The present qualitative analysis allowed a sampling of a group of professionals regarding their work, experiences, values, and beliefs about health care, as well as the ethical dilemmas they face. A larger study will be conducted using this analysis, and based upon these data and our interpretive findings.

At present, the study revealed that life history values are closely related to those in the specific field of medicine. Life history values give the physician (and other healthcare workers), a “local” cultural competence that allows interaction with those in the community they treat, and we believe that it is possible to fine tune the clinical virtues from this “local” cultural competence. However, the latter is not sufficient for interactions with patients of different cultural backgrounds; hence, additional work is needed to incorporate the required intercultural values in the clinical professions [32]. The values of healthcare professionals reveal their priorities, how they administer medical assistance, and the institutional relationships established in the work place. These factors certainly impact on the clinical relationship and on the clinical and ethical decisions of cardiology. An important goal therefore, is to engage both clinicians’ and patients’ values in ethical discernment and judgment, in order to establish and fortify a closer and sounder clinical relationship between the physician and patient.

To achieve this, we posit that it is necessary to fulfill three established roles of healthcare professionals [28]: provider of medical assistance, guidance counselor, and educator. Our results show that the most established roles in this group of professionals are those of providers of medical assistance and guidance, but the role of educator is less well articulated.

The role of provider of medical assistance is central to evidence-based medicine and this is reflected in the wide use of epistemic values related to patient treatment, such as the proper collection of clinical data, effective diagnosis, and efficiency of treatment. The predominance of this role is related to the life history values linked to authority and achievement. The roles of guidance counselor and educator are more intrinsically related to VBM, but they are also key for therapeutic success in EBM since therapeutic adherence and cost-effectiveness of treatment depend at least to some extent, upon an informed patient, who- through a clinicians’ deliberation and actions- is an active rather than passive participant in the clinical encounter [33]. This would clearly support how EBM and VBM are vital to strengthen the goals of the medical practice.

The absence of the educator role indicates that not enough time is dedicated to building communication, and generating trust between the medical personnel and the patient. Through the role of educator, the patient is made co-responsible for the success of his treatment and the prognosis of his illness [34,35]. The development of this role provides opportunities for primary prevention by modifying lifestyles. In general, the field of cardiology has tended to pay less attention to the role of educator, despite the need to prevent cardiovascular disease and the evident importance of the physician in advocating, developing, and guiding new lifestyle patterns for their patients. Patient autonomy is related to this role of the medical professional [36], however, this was rarely mentioned by the interviewees.

Respect for the patients’ autonomy sustains a view of the patient as person with her own values, life plans, and decision-making ability. In order to promote patient autonomy, healthcare personnel must develop cultural competences and skills for the ethical discernment in multicultural contexts [18]. Respecting patient autonomy may require changes in physicians’ beliefs and behaviors as well as change in the institutional environment, and in light of this it was interesting that conservation values were dominant and more frequent than those of openness to change [27].

In the cardiology professionals we studied, there is a clear concern for justice, tolerance, and equal access to healthcare. Justice was revealed as central in all fields of ethical discernment, which suggests a strong commitment to service. The life history values closely linked to justice are benevolence and universality, which were prioritized by the interviewees in their personal development. Other virtues related to justice are respect, compassion, trust, temperance, and patience. These values strengthen the roles of guidance counselor and educator; therefore their promotion could stimulate the development of the educator role that is absent to this day.

The qualitative analysis conducted in this study allowed for interpretations of the values present in medical personnel, but it remains difficult to establish strategies to improve healthcare systems based only on the present sample. As such, a nationwide study is required.

In conclusion, we hold that the ends of medical practice (curing, healing and caring) encompass both EBM and VBM [18,37]; and this necessitates the simultaneous fulfillment of the three roles of the healthcare professional. Our work supports that clinical practice values are closely related to those of life history. This is important when developing the ethical formation of medical students, and healthcare employees. As well, we believe that knowledge of the axiology of healthcare professions can be applied to the selection of candidates who seek to study and pursue these careers.

These findings could provide a preliminary framework from which to identify, develop and evaluate educational strategies and the best clinical practices. Clearly, further larger scale study is required, but we maintain that if the present findings are replicated at such a scale, it would indicate the need for a better conformity of values, virtues and evidence in the grounding of medicine to cultural competence [18]. This may be a starting point for the modification of institutional regulations to strengthen the pairing of evidence-based medicine and value-based medicine.

## Endnotes

^a^ According to the New York Heart Association (NYHA), this refers to a mild cardiac insufficiency where any physical activity gives rise to fatigue, palpitations, or dyspnea.

^b^ Representations are learned as a system that presents dilemmas, conflicts and even contradictions, but it constitutes a system of expectations for clinical intervention, in practice is where these representations are realized and modified [38].

## Abbreviations

EBM: Evidence based medicine; VBM: Value based medicine; EVAT: Escala de Valores en el Trabajo (Work Value Scale).

## Competing interests

The authors declare no competing interests.

## Authors’ contributions

Conception and design: MMAB, NAB. Collection and assembly of the data: MMAB, SR, RND, JM, CC. Data analysis and interpretation: MMAB, NAB, AdH, AS, RND, JM, CC, SR, CC, CMO Drafting of the article: MMA, NAB, AdH, RND. Critical revision of the article for important intellectual content: DB, CMO, HC, MMAB, NAB, AdeH, AS, RND, JM, CC, SR. Final approval of the article: all authors. Obtaining of funding: MMAB. All authors read and approved the final manuscript.

## Authors’ information

Adalberto de Hoyos: Grupo transfuncional en ética clínica, Centro Médico Nacional Siglo XXI, IMSS, México, FES Acatlán, Universidad Nacional Autónoma de México, Mexico. Rodrigo Nava-Diosdado: Grupo transfuncional en ética clínica, Centro Médico Nacional Siglo XXI, IMSS, México.Jorge Mendez. Grupo transfuncional en ética clínica, Centro Médico Nacional Siglo XXI, IMSS, México. Sergio Ricco: Universidad Pedagógica Nacional, Mexico. Ana Serrano: Grupo transfuncional en ética clínica, Centro Médico Nacional Siglo XXI, IMSS, México. Carmen Cisneros: Instituto Nacional de Perinatología. Carlos Macías-Ojeda: Grupo transfuncional en ética clínica, Centro Médico Nacional Siglo XXI, IMSS, México. Universidad Anáhuac. Héctor Cisneros: Facultad de Ciencias, UNAM, Mexico. David Bialostozky: Grupo transfuncional en ética clínica, Centro Médico Nacional Siglo XXI, IMSS, México. Nelly Altamirano-Bustamante: Grupo transfuncional en ética clínica, Centro Médico Nacional Siglo XXI, IMSS, México, Instituto Nacional de Pediatría, Secretaría de Salud, Mexico. Myriam M. Altamirano-Bustamante: Grupo transfuncional en ética clínica, Centro Médico Nacional Siglo XXI, IMSS, México Unidad de Investigación de Enfermedades Metabólicas, Centro Médico Nacional Siglo XXI, IMSS.
